# Assessment of the Surgical Oncology Case Volume Within the Public Sector in Tanzania

**DOI:** 10.1200/GO.23.00316

**Published:** 2024-03-07

**Authors:** Nathan R. Brand, Larry Akoko, Vihar Kotecha, Theresia Mwakyembe, Masumbuko Mwashambwa, Rukia Hamid, Deo Hando, Charles Komba, Ally Mwanga, Peter Mbele, Paul Itule, Joshua Jackson, Mungeni Misidai, Cameron Gaskill, Doruk Ozgediz

**Affiliations:** ^1^ Department of Surgery, University of California, San Francisco, San Francisco, CA; ^2^ UCSF's Center for Health Equity in Surgery and Anesthesia, San Francisco, CA; ^3^ Department of Surgery, Muhimbili University for Health and Allied Sciences, Dar es Salaam, Tanzania; ^4^ Department of Surgery, Catholic University for Health and Allied Sciences, Mwanza, Tanzania; ^5^ Department of Surgery, Kilimanjaro Christian Medical Center, Moshi, Tanzania; ^6^ Department of Surgery, University of Dodoma, Dodoma, Tanzania; ^7^ Department of Surgery, Mnazi Mmoja Hospital, Zanzibar, Tanzania; ^8^ Department of Surgery, Mbeya Zonal Referral Hospital, Mbeya, Tanzania; ^9^ Department of Surgery, Muhimbili National Hospital, Dar es Salaam, Tanzania; ^10^ Department of Surgery, Dodoma Regional Referral Hospital, Dodoma, Tanzania; ^11^ Department of Surgery, Sekou Toure Regional Referral Hospital, Mwanza, Tanzania; ^12^ Department of Urology, Tanga Regional Referral Hospital, Tanga, Tanzania; ^13^ Department of Surgery, University of California, Davis, Sacramento, CA

## Abstract

**PURPOSE:**

Surgery provides vital services to diagnose, treat, and palliate patients suffering from malignancies. However, despite its importance, there is little information on the delivery of surgical oncology services in Tanzania.

**METHODS:**

Operative logbooks were reviewed at all national referral hospitals that offer surgery, all zonal referral hospitals in Mainland Tanganyika and Zanzibar, and a convenience sampling of regional referral hospitals in 2022. Cancer cases were identified by postoperative diagnosis and deidentified data were abstracted for each cancer surgery. The proportion of the procedures conducted for patients with cancer and the total number of cancer surgeries done within the public sector were calculated and compared with a previously published estimate of the surgical oncology need for the country.

**RESULTS:**

In total, 69,195 operations were reviewed at 10 hospitals, including two national referral hospitals, five zonal referral hospitals, and three regional referral hospitals. Of the cases reviewed, 4,248 (6.1%) were for the treatment of cancer. We estimate that 4,938 cancer surgeries occurred in the public sector in Tanzania accounting for operations conducted at hospitals not included in our study. Prostate, breast, head and neck, esophageal, and bladder cancers were the five most common diagnoses. Although 387 (83%) of all breast cancer procedures were done with curative intent, 506 (87%) of patients with prostate and 273 (81%) of patients with esophageal cancer underwent palliative surgery.

**CONCLUSION:**

In this comprehensive assessment of surgical oncology service delivery in Tanzania, we identified 4,248 cancer surgeries and estimate that 4,938 likely occurred in 2022. This represents only 25% of the estimated 19,726 cancer surgeries that are annually needed in Tanzania. These results highlight the need to identify strategies for increasing surgical oncology capacity in the country.

## Teaser

Assessment of surgical outputs in Tanzania finds the country is conducting 25% of the necessary cancer surgeries.

## INTRODUCTION

Globally, it is estimated that there were 19.3 million new cancer cases and 10 million cancer-related deaths in 2020. Of these, 1.1 million cases and 720,000 deaths took place in Africa.^1^ This burden of disease is expected to continue to grow, and it is estimated that the incidence of cancer will double in Africa by 2040.^2^ In Tanzania specifically, a country in East Africa with a population of 65 million, cancer is the fifth leading cause of death among adults. It is estimated that 40,464 new cancer cases and 26,945 cancer-related deaths occur annually.^1,3^

**CONTEXT**

**Key Objective**
How many and what types of cancer surgeries occur each year in Tanzania?
**Knowledge Generated**
We estimated that in 2022, 4,938 cancer surgeries occurred in Tanzania, with a majority being for prostate, breast, head and neck, esophageal, and bladder cancers. Surgeries for breast cancer were primarily for curative intent; however, most of the surgeries for prostate and esophageal cancers were palliative.
**Relevance**
Tanzania is conducting only 25% of the estimated 19,725 cancer surgeries that are needed. This baseline information is integral for both measuring future interventions to improve surgical oncology capacity, and to highlight the need for additional strategies to promote the diagnosis and treatment of patients with curable disease.

Surgery provides vital services to diagnose, treat, and palliate patients suffering from malignancies and it is estimated that 80% of all patients with cancer will require surgery at some point during their disease.^4,5^ However, in low- and middle-income countries (LMICs), these services are often under-resourced and only available at the tertiary level. In 2015, the Lancet launched a Commission on Global Surgery. This was one of the first large-scale efforts to highlight the profound need to increase surgical capacity in LMICs, and it estimated that globally, 5 billion people lack access to safe surgical care.^6^ Subsequently, there have been significant efforts to increase surgical capacity and improve access to safe surgery globally. The Commission proposed population-level metrics for surgical care and emphasized underinvestment and value. Simultaneously, the Disease Control Priorities Project proposed a package of essential surgical interventions.^7^ The complexity of surgical oncology care complicates the rapid expansion of surgical services for patients with cancer in LMICs as the surgical need is increasing. Recently, the Lancet Commission on Global Surgical Oncology estimated that by 2030, there will be 40 million cancer surgeries that will need to be performed annually and that most of these patients will be living in LMICs.^8^

As previously mentioned, the incidence of cancer is expected to rise dramatically in LMICs. In sub-Saharan Africa specifically, it is expected that by 2040, 1.4 million people annually will die of cancer, representing a 106% increase from the current incidence.^9^ To prepare for the cancer epidemic that is expected to affect Tanzania, a country of 65 million in Eastern Africa, a thorough understanding of the current surgical oncology landscape in the country is necessary. This benchmark can be used as a baseline to assess the impact of future interventions on surgical oncology care, to identify centers of expertise where local training programs may be developed, and to begin to convince health care facilities in Tanzania to focus on surgical care of oncology patients. There have been no previous national assessments of surgical oncology service delivery, and this study aimed to address this gap.

## METHODS

### Study Design and Setting

This was a hospital-based retrospective cross-sectional study undertaken in Tanzania, a lower-middle–income country in East Africa. The study focused on the busiest hospitals that provide most of the surgical care in the country. This was determined by literature review, and discussions with the Tanzania Surgical Association and the Ministry of Health.^10^ All the hospitals identified were either public or faith-based. In Tanzania, hospitals are organized in a pyramid-like hierarchy. Most common are dispensaries followed by health centers. The 85 district hospitals are the lowest-level hospital on the pyramid that is expected to provide emergency surgical services. This is followed by regional hospitals, which primarily provide emergency surgical care with some elective procedures as well. In Tanzania, there are 18 regional hospitals that provide surgical care.^11^ The five zonal hospitals provide most of the elective surgical care in Tanzania, followed by the two specialized national referral hospitals.^11^ For this study, all national referral hospitals that provide surgical care were included (Muhimbili National Hospital and Muhimbili Orthopaedic Institute). In addition, we included all zonal referral hospitals on mainland Tanganyka (Bugando Medical Centre, Kilimanjaro Christian Medical Centre, Mbeya Zonal Referral Hospital, and Benjamin Mkapa Hospital) and the referral hospital for Zanzibar (Mnazi Moja Hospital). Finally regional hospitals were included using convenience sampling by contacting busy regional referral hospitals identified by leadership at the Tanzanian Surgical Association and the Ministry of Health and including those who were amendable to participation. In total, three regional referral hospitals were contacted and three were included (Dodoma Regional Referral Hospital in the country's capital city, Tanga Regional Referral Hospital in the northern coast, and Sekou Toure Regional Referral Hospital in Mwanza on Lake Victoria). To estimate the cancer cases that occurred at the 15 regional hospitals that did not participate in this study, we averaged the number of cancer cases that occurred at the three included regional hospitals to calculate an estimated annual cancer case volume for each regional hospital. We then compared the total number of cancer cases that occurred during 2022 with the estimated number of cancer surgeries that are needed to treat every patient with cancer in Tanzania, 19,726.^12^

### Data Collection and Analysis

Data were abstracted from January 1, 2022, to December 31, 2022, using the operative logbook at each hospital. Previous studies in East Africa have shown that these logbooks capture more than 90% of all surgeries that take place.^13^ At Muhimbili Orthopaedic Institute, Bugando Medical Centre, Kilimanjaro Christian Medical Centre, and Benjamin Mkapa Hospital, these case logs are electronic and are filled by the surgeon after completion of a surgery. At Muhimbili National Hospital, Mbeya Zonal Regional Hospital, Dodoma Regional Referral Hospital, Tanga Regional Referral Hospital, and Mnazi Moja Hospital, these logbooks are paper-based and were reviewed by the study team (N.R.B., R.H., D.H., P.L., J.J., M.M., and P.M.). Cancer cases were identified using the postoperative diagnosis. Date of service, age, sex, postoperative diagnosis, and surgical procedure were abstracted for all cancer cases. Surgeries that seemed unrelated to a cancer diagnosis, and cases that were missing either the type of surgery or the postoperative diagnosis were not included in the final database. In total, 253 (6%) of the cancer cases identified were excluded. Abstracted data were uploaded into REDCAP kept on the Muhimbili University of Health and Allied Sciences web server and analyzed using STATA SE version 17 (StataCorp LLC, College Station, TX). Data analysis included summary statistics and multivariate logistic regression analysis.

### Ethical Clearance

The study was approved by the institutional review boards (IRBs) in Tanzania at Muhimbili University of Health and Allied Sciences, MUHAS-REC-08-2022-1324, the National Institute of Medical Research, NIMR/HQ/R.8a/Vol.IX/4209, and the Zanzibar Health Research Institute, ZAHREC/03/REC/MAR/2023/0. In addition, letters of permission were provided by all hospitals where data collection took place by the hospital administration. N.R.B., who conducted most of the data collection, was also registered with the Tanzanian Commission for Science and Technology, COSTECH. In the United States, the University of California, San Francisco's IRB deemed the protocol exempt, 22-37294.

## RESULTS

**FIG 1:**
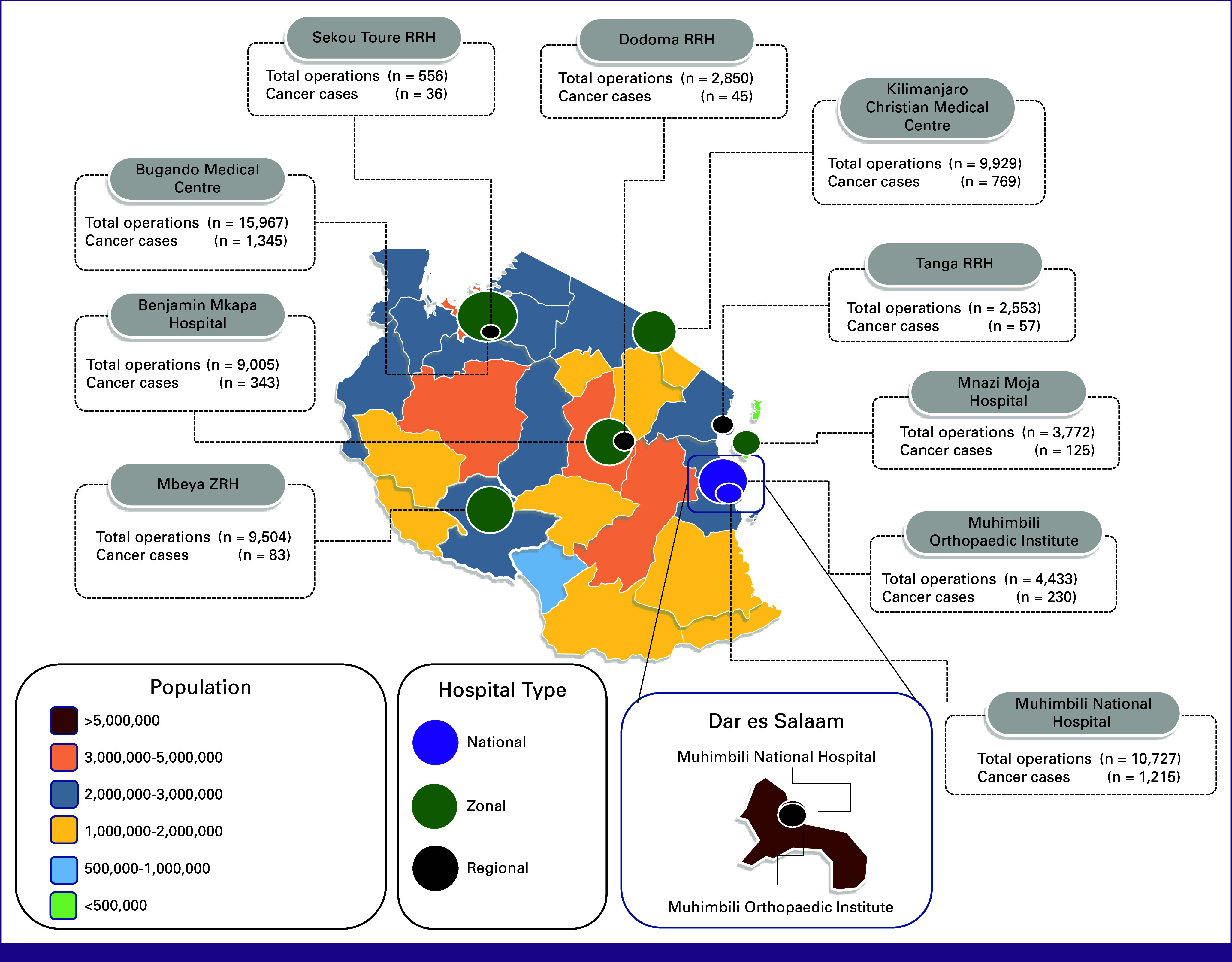
Location of each participating hospital, number of cancer procedures completed, and population by region in 2022. This figure shows the location of all participating hospitals color coded by hospital type over a heat map showing the population of Tanzania by region. Size of hospital circle corresponds to the total number of annual operations completed in 2022. RRH, regional referral hospital; ZRH, zonal referral hospital.

In total, 69,195 operations were reviewed at 10 hospitals throughout Tanzania during 2022. Of the 69,195 operative cases that were reviewed, 4,248 oncology cases were identified and abstracted for analysis, including 1,776 (42%) surgeries with curative intent and 1,145 (27%) surgeries for palliation. Figure 1 shows the geographic location of all hospitals included in the study and the population of each region on the basis of the 2022 census.^14^

In total, Tanzania has 18 regional referral hospitals that provide surgical services in the country.^11^ On the basis of the three regional hospitals that were included in our study, each regional hospital conducts on average 46 cancer cases each year. By extrapolating this case volume to the 15 regional hospitals not included in the study, there were an estimated additional 690 cancer surgeries conducted in the public sector and not included in our study. Therefore, in total, we estimate that 4,938 cancer surgeries occurred within the public sector in 2022 in Tanzania. This accounts for 25% of the 19,726 cancer cases that are estimated to be needed to treat all patients with cancer in Tanzania.^12^

Of the abstracted cancer surgeries, 3,201 (75%) had information on sex with a male predominance at 1,674 (52%). Cancer diagnoses spanned across all age groups. Among adults, the median age was 54 years, and children, defined in Tanzania as patients age 12 years or younger, made up 7.9% of the cancer surgeries. The five most common cancer sites among adults were prostate (583, 13.8%), breast (465, 11.0%), head and neck (435, 10.2%), esophageal (337, 7.9%), and bladder (314, 7.4%). For children, the five most common cancer sites were eye (94, 28.7%), kidney (60, 18.4%), central nervous system (43, 12.8%), bone (28, 8.4%), and head and neck (24, 7.2%).

**FIG 2:**
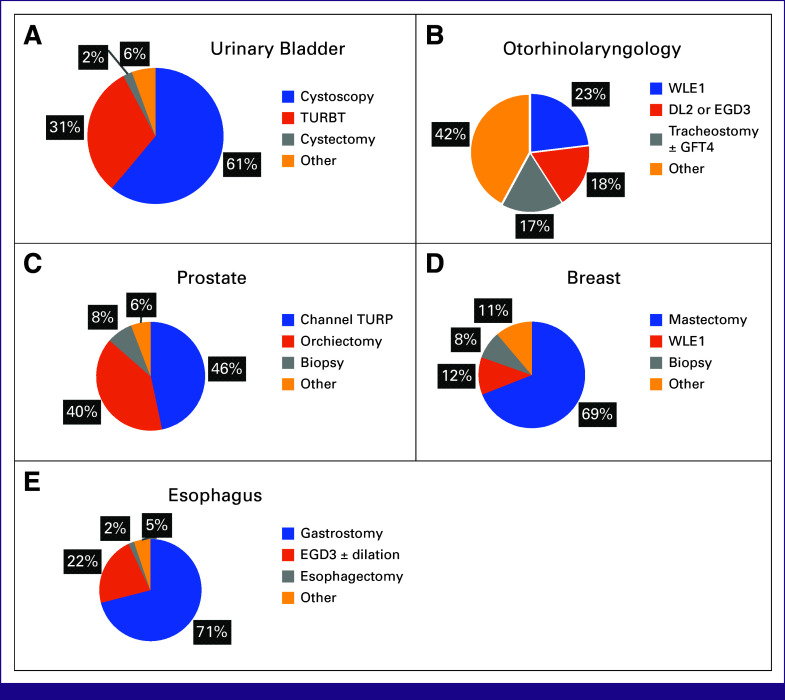
Operative procedure distribution for top five cancers: (A) urinary bladder, (B) otorhinolaryngology, (C) prostate, (D) breast, and (E) esophagus. DL, direct laryngoscopy; EGD, esophagoduodenoscopy; GFT, gastrostomy feeding tube; TURBT, transurethral resection of bladder tumor; TURP, transurethral resection of the prostate; WLE, wide local excision.

Table 1 describes the operative intent of the most common cancer diagnosis for all ages. In total, the most performed cancer surgeries were biopsy (590, 13.9%), excision (544, 12.8%), modified radical mastectomy (324, 7.6%), feeding gastrostomy (303, 7.1%), and channel transurethral resection of prostate (272, 6.4%). Because a laparotomy can be diagnostic, curative, or palliative, cases listed as laparotomy in the case log were not further categorized by treatment intent but kept as separate category. Table 2 describes the most common procedures for patients with cancer both overall and by hospital type in Tanzania. In Figure 2, we report the most common procedures conducted for prostate, breast, esophageal, head and neck, and bladder cancers.

**TABLE 1:** Most Common Cancer Site by Surgical Intent

Cancer Site	Total, No.	Diagnosis, No. (%)	Treatment, No. (%)	Palliation, No. (%)	Reconstruction, No. (%)	Laparotomy, No. (%)
All sites	4,248	1,082 (25)	1,776 (42)	1,145 (27)	82 (2)	163 (4)
Prostate	583	68 (11.5)	6 (1.0)	506 (87.0)	0 (0)	3 (0.5)
Breast	465	66 (14.2)	387 (83.2)	9 (2.0)	3 (0.6)	0 (0)
Head and neck	435	147 (33.8)	202 (46.4)	77 (17.7)	7 (1.6)	2 (0.5)
Esophageal	337	55 (16.3)	6 (1.8)	273 (81.0)	0 (0)	3 (1.0)
Bladder	314	193 (61.5)	101 (32.2)^a^	13 (4.1)	3 (0.9)	4 (1.3)
Colorectal	255	66 (25.9)	74 (29.0)	56 (22.0)	16 (6.3)	43 (16.9)
Skin	238	54 (22.7)	155 (65.1)	0 (0)	29 (12.2)	0 (0)
Brain/central CNS	190	20 (10.5)	130 (68.5)	39 (20.5)	1 (0.5)	0 (0)
Eye	188	87 (46.3)	99 (52.7)	2 (1.0)	0 (0)	0 (0)
Soft tissue/sarcoma	183	36 (19.7)	135 (73.8)	1 (0.5)	6 (3.3)	5 (2.7)
Bone	166	82 (49.4)	58 (34.9)	20 (12.1)	6 (3.6)	0 (0)
Cervix	129	28 (21.7)	83 (62.0)	17 (13.1)	2 (1.6)	2 (1.6)
Ovary	111	12 (10.8)	53 (47.8)	25 (22.5)	1 (0.9)	20 (18.0)
Kidney	104	3 (2.9)	93 (89.4)	3 (2.9)	0 (0)	5 (4.8)
Stomach	101	19 (18.8)	40 (39.6)	26 (25.7)	0 (0)	16 (15.8)
Unknown primary	80	27 (33.7)	20 (25.0)	5 (6.3)	0 (0)	28 (35.0)
Liver/gallbladder/pancreas	75	18 (24.0)	11 (14.7)	31 (41.3)	0 (0)	15 (20.0)
Uterine	65	8 (12.3)	46 (70.8)	6 (9.2)	0 (0)	5 (7.7)
Thyroid	43	3 (7.0)	36 (83.7)	4 (9.3)	0 (0)	0 (0)
Other	186	90 (48.4)	44 (23.6)	32 (17.2)	8 (4.3)	12 (6.5)

Abbreviation: TURBT, trans urethral resection of bladder tumor.

^a^ All TURBT were coded as an operation for treatment.

**TABLE 2:** Most Common Oncologic Procedures by Hospital Type

Surgical Procedure	Total, No.	National Hospital, No. (%)	Zonal Hospital, No. (%)	Regional Hospital, No. (%)
Total procedures	4,248	1,445 (34.0)	2,665 (62.7)	138 (3.3)
EUA/biopsy	590	293 (49.7)	237 (40.2)	60 (10.1)
Excision	544	148 (27.2)	383 (70.4)	13 (2.4)
Mastectomy	324	115 (35.5)	194 (59.9)	15 (4.6)
Feeding gastrostomy	303	112 (37.0)	188 (62.0)	3 (1.0)
Channel TURP	272	42 (14.4)	230 (84.6)	0 (0)
Orchidectomy	238	71 (29.8)	164 (68.9)	3 (1.3)
Cystoscopy	214	100 (46.7)	114 (53.3)	0 (0)
Hysterectomy ± BSO	182	38 (20.9)	128 (70.3)	16 (8.8)
Laparotomy	162	45 (27.8)	110 (67.9)	7 (4.3)
EGD	114	7 (6.1)	107 (93.9)	0 (0)
TURBT	103	12 (11.6)	91 (88.4)	0 (0)
Craniotomy	99	86 (86.9)	13 (13.1)	0 (0)
Nephrectomy	94	44 (46.8)	50 (53.2)	0 (0)
Colostomy	81	30 (37.0)	48 (59.3)	3 (3.7)
Laryngoscopy	68	41 (60.3)	27 (39.7)	0 (0)
Colectomy	57	22 (38.6)	34 (59.6)	1 (1.8)
Amputation	51	16 (31.4)	28 (54.9)	7 (13.7)
Tracheostomy	51	3 (5.9)	47 (92.1)	1 (2.0)
Palliative debulking	46	10 (21.8)	33 (71.7)	3 (6.5)
Gastrectomy	38	8 (21.0)	30 (79.0)	0 (0)
Thyroidectomy	37	19 (51.4)	18 (48.6)	0 (0)
Microlaryngeal surgery	36	0 (0)	36 (100)	0 (0)
Enucleation of the eye	35	18 (51.4)	16 (45.7)	1 (2.9)
Skin graft	34	1 (2.9)	33 (97.1)	0 (0)
Parotidectomy	28	16 (57.1)	12 (42.9)	0 (0)
Biliary bypass	26	14 (53.8)	12 (46.2)	0 (0)
Lumpectomy	21	2 (9.5)	16 (76.2)	3 (14.3)
VP shunt	18	6 (33.3)	12 (66.7)	0 (0)
Neck dissection	17	3 (17.7)	14 (82.3)	0 (0)
Gastric bypass	15	11 (73.3)	4 (26.7)	0 (0)
Laparoscopy	14	0 (0)	14 (100)	0 (0)
Cystectomy	14	2 (14.3)	12 (85.7)	0 (0)
Cholecystectomy	12	0 (0)	12 (100)	0 (0)
Tonsillectomy	11	1 (9.0)	10 (91.0)	0 (0)
APR	10	8 (80)	2 (20)	0 (0)
Low anterior resection	8	2 (25)	6 (75)	0 (0)
Laryngectomy	8	1 (12.5)	7 (87.6)	0 (0)
Thoracotomy	7	7 (100)	0 (0)	0 (0)
Esophagectomy	6	2 (33.3)	4 (66.7)	0 (0)
Lung resection	5	0 (0)	5 (100)	0 (0)
Radical prostatectomy	5	3 (60)	2 (40)	0 (0)
Whipple procedure	4	1 (25.0)	3 (75.0)	0 (0)
Adrenalectomy	4	4 (100)	0 (0)	0 (0)
Cystostomy	3	2 (66.7)	1 (33.3)	0 (0)
Splenectomy	2	1 (50.0)	1 (50.0)	0 (0)
Hepatectomy	2	2 (100)	0 (0)	0 (0)
LEEP	2	0 (0)	2 (100)	0 (0)
Other	233	76 (32.6)	155 (66.5)	2 (0.9)

Abbreviations: APR, abdominalperoneal resection; BSO, bilateral salpingo-oophorectomy; EGD, esophagoduodenoscopy; EUA, exam under anesthesia; LEEP, loop electrosurgical excision procedure; TURBT, transurethral resection of bladder tumor; TURP, transurethral resection of the prostate; VP, ventriculoperitoneal.

Finally, we conducted three multiple logistic regression analyses to identify variables associated with undergoing cancer surgery for curative, diagnostic, and palliative intent. All models included hospital type, patients older than 60 years, and the five most common cancer diagnoses as covariates. Overall, breast cancer and being younger than 60 years were associated with undergoing a procedure for curative intent, and having prostate, esophageal, or head and neck cancers, and being older than 60 years were associated with undergoing palliative surgery (Table 3).

**TABLE 3:** Association of Surgical Intent With Cancer Type

Factor	Odds Ratio (95% CI)	*P*
Curative intent		
Breast cancer	4.84 (3.73 to 6.27)	<.0001
Less than 60 years	1.24 (1.07 to 1.45)	.005
Prostate cancer	0.01 (0.005 to 0.026)	<.0001
Esophageal cancer	0.02 (0.008 to 0.041)	<.0001
Bladder cancer	0.48 (0.37 to 0.62)	<.0001
Head and neck cancer	0.88 (0.71 to 1.09)	.2
Hospital type^a^	1.04 (0.92 to 1.19)	.5
Diagnostic intent		
Bladder cancer	4.6 (3.59 to 5.93)	<.0001
Head and neck cancer	1.48 (1.19 to 1.86)	.001
Hospital type^a^	1.17 (1.03 to 1.35)	.02
Prostate cancer	0.40 (0.30 to 0.54)	<.0001
Breast cancer	0.48 (0.36 to 0.63)	<.0001
Esophageal cancer	0.57 (0.42 to 0.77)	<.0001
More than 60 years	0.92 (0.78 to 1.07)	.3
Palliative intent		
Prostate cancer	38.0 (28.3 to 51.2)	<.0001
Esophageal cancer	28.0 (20.7 to 37.8)	<.0001
More than 60 years	1.36 (1.11 to 1.67)	.003
Head and neck cancer	1.39 (1.05 to 1.85)	.02
Bladder cancer	0.28 (0.16 to 0.49)	<.0001
Breast cancer	0.14 (0.07 to 0.27)	<.0001
Hospital type^a^	1.03 (0.85 to 1.23)	.8

^a^ Regional hospital = 1, zonal hospital = 2, national hospital = 3.

## DISCUSSION

In this study, we estimate the surgical oncology case volume, distribution, and intent at all the national referral hospitals that offer surgical care, all the zonal referral hospitals, and a convenience sampling of regional referral hospitals in Tanzania. This represents the most comprehensive reported assessment of surgical oncology output. We estimate, that in total, 4,938 cancer procedures were done in the public sector in Tanzania. This includes the estimated 690 cases that were done at the 15 regional referral hospitals that did not participate in this study. This represents only 25% of the estimated 19,726 cancer surgeries that were estimated to be needed annually in Tanzania on the basis of the current population and incidence of cancer.^12^ In the only other similar study in sub-Saharan Africa, 21% of the necessary cancer cases were done in Ghana (2021) using New Zealand as a benchmark for the number of surgeries necessary per cancer case.^15^ The causes for the high unmet need for surgical oncology care within the public sector in Tanzania are multifaceted and include barriers such as late-stage diagnosis, financial strain, and limited geographic access. Although these factors were not within the scope of this research study, they affect the country's ability to provide cancer surgery in the country and may be a focus of future work of this group.^16-20^

Of the abstracted cancer procedures, only 1,776 (equivalent to two of five) of the cases were with curative intent, including 387 surgeries for women with breast cancer. However, there is an estimated 2,261 new cases of breast cancer that occur each year in Tanzania, suggesting that only 17% of these women are accessing curative surgical services within the public sector in Tanzania.^21^ Reviews of patients with breast cancer at Kilimanjaro Christina Medical Centre, however, showed that only 17% of patients with breast cancer present with stage IV disease, suggesting that there is a significant population of women with curable breast cancer unable to access potentially curative surgical care in Tanzania.^18^ In addition, the high proportion of patients with prostate and esophageal cancers that underwent palliative surgery highlights the need to identify patients with cancer early when surgical resection is still an option. The high proportion of palliative cases for esophageal and prostate cancers is consistent with previous series from East Africa which describe frequent late-stage diagnosis when surgical treatment options are limited to palliation.^22,23^

Some cancers with high prevalence in Tanzania are commonly operated on such as breast, esophageal, and prostate cancers, while others, such as cervical cancer, are highly prevalent in Tanzania but rarely operated on in the country. This is likely because screening and diagnosis can be conducted in a procedure room rather than a formal operating theater and the many barriers that prevent patients with cervical cancer from accessing care. These findings are consistent with a recent prospective study of patients with cervical cancer at Mbeya that found that only 8% of patients diagnosed with cervical cancer receive surgery.^24^ The results of this study highlight the need to identify and address barriers that are preventing patients with cervical cancer from accessing surgical services in Tanzania.

There are several limitations to be noted in our study. First, cancer diagnosis was made via the postoperative diagnosis in the surgical logbook and was not able to be verified with a pathologic diagnosis. In addition, the data that we were able to abstract were limited to the few variables regularly collected in all operative logbooks, making it impossible to fully evaluate the oncologic quality of the surgery provided or the clinical course of patients included in study. However, the use of this data source allowed us to collect significant data from the busiest hospitals across Tanzania. Given these limitations, the findings of this study should be seen as an estimation of the surgical oncology landscape in Tanzania rather than definitive assessment. However, as an estimation, our study provides important baseline of surgical oncology case volume for the country, which can be used by policymakers, clinicians, and researchers in the country looking to understand the current landscape of cancer surgery in the country.

Our study provides the most thorough assessment of surgical oncology output within the public health sector in Tanzania. We estimate that currently, Tanzania's public health sector is providing 25% of the cancer surgery they need, given their current population and cancer incidence. Of these cases, less than half are for curative intent. As well, we show that patients with esophageal and prostate cancers primarily receive palliative surgery and that only 18% of patients with breast cancer are accessing curative surgery. This study provides an invaluable baseline assessment of the cancer surgery landscape for Tanzania and is a call to action to improve both the number of patients with cancer able to access both surgical services and curative treatment in the country.

## References

[1] Sung H, Ferlay J, Siegel RL, et al: Global cancer statistics 2020: GLOBOCAN estimates of incidence and mortality worldwide for 36 cancers in 185 countries. CA Cancer J Clin 71:209-249, 2021 33538338 10.3322/caac.21660

[2] Vanderpuye V, Hammad N, Martei Y, et al: Cancer care workforce in Africa: Perspectives from a global survey. Infect Agent Cancer 14:11, 2019 31139248 10.1186/s13027-019-0227-8 PMC6528232

[3] Mboera LEG, Rumisha SF, Lyimo EP, et al: Cause-specific mortality patterns among hospital deaths in Tanzania, 2006-2015. PLoS One 13:e0205833, 2018 30379899 10.1371/journal.pone.0205833 PMC6209209

[4] Hoekstra HJ, Wobbes T, Heineman E, et al: Fighting global disparities in cancer care: A surgical oncology view. Ann Surg Oncol 23:2131-2136, 2016 27038459 10.1245/s10434-016-5194-3 PMC4889619

[5] Zogg CK: Recognition of surgical need as part of cancer control in Africa. Lancet Oncol 14:e289, 2013 10.1016/S1470-2045(13)70210-9 23816291

[6] Meara JG, Leather AJM, Hagander L, et al: Global surgery 2030: Evidence and solutions for achieving health, welfare, and economic development. Lancet 386:569-624, 2015 25924834 10.1016/S0140-6736(15)60160-X

[7] Mock CN, Donkor P, Gawande A, et al: Essential surgery: Key messages from Disease Control Priorities, 3rd edition. Lancet 385:2209-2219, 2015 25662414 10.1016/S0140-6736(15)60091-5 PMC7004823

[8] Sullivan R, Alatise OI, Anderson BO, et al: Global cancer surgery: Delivering safe, affordable, and timely cancer surgery. Lancet Oncol 16:1193-1224, 2015 26427363 10.1016/S1470-2045(15)00223-5

[9] Ngwa W, Addai BW, Adewole I, et al: Cancer in sub-Saharan Africa: A Lancet Oncology Commission. Lancet Oncol 23:e251-e312, 2022 35550267 10.1016/S1470-2045(21)00720-8 PMC9393090

[10] Nyberger K, Jumbam DT, Dahm J, et al: The situation of safe surgery and anaesthesia in Tanzania: A systematic review. World J Surg 43:24-35, 2019 30128771 10.1007/s00268-018-4767-7 PMC6313359

[11] National Surgical, Obstetric and Anaesthesia Plan (NSOAP) 2018-2025. The United Republic of Tanzania. https://www.pgssc.org/_files/ugd/d9a674_4daa353b73064f70ab6a53a96bb84ace.pdf

[12] Zafar SN, Siddiqui AH, Channa R, et al: Estimating the global demand and delivery of cancer surgery. World J Surg 43:2203-2210, 2019 31115586 10.1007/s00268-019-05035-6

[13] Anderson GA, Ilcisin L, Abesiga L, et al: Surgical volume and postoperative mortality rate at a referral hospital in Western Uganda: Measuring the Lancet Commission on Global Surgery indicators in low-resource settings. Surgery 161:1710-1719, 2017 28259351 10.1016/j.surg.2017.01.009

[14] Tanzania Census: Tanzania census dissemination platform. Tanzania census information. https://sensa.nbs.go.tz/

[15] Gaskill CE, Gyedu A, Stewart B, et al: Improving global surgical oncology benchmarks: Defining the unmet need for cancer surgery in Ghana. World J Surg 45:2661-2669, 2021 34152449 10.1007/s00268-021-06197-y

[16] Rick TJ, Aagard M, Erwin E, et al: Barriers to cancer care in Northern Tanzania: Patient and health-system predictors for delayed presentation. JCO Glob Oncol 10.1200/GO.21.00253 10.1200/GO.21.00253 PMC8791818 34665667

[17] Chalya PL, Mchembe MD, Mabula JB, et al: Clinicopathological patterns and challenges of management of colorectal cancer in a resource-limited setting: A Tanzanian experience. World J Surg Oncol 11:88, 2013 23597032 10.1186/1477-7819-11-88 PMC3637367

[18] Gnanamuttupulle M, Henke O, Ntundu SH, et al: Clinicopathological characteristics of breast cancer patients from Northern Tanzania: Common aspects of late stage presentation and triple negative breast cancer. Ecancermedicalscience 15:1282, 2021 34824605 10.3332/ecancer.2021.1282 PMC8580599

[19] Otieno ES, Micheni JN, Kimende SK, et al: Provider delay in the diagnosis and initiation of definitive treatment for breast cancer patients. East Afr Med J 87:143-146, 2010 23057288 10.4314/eamj.v87i4.62201

[20] Otieno ES, Micheni JN, Kimende SK, et al: Delayed presentation of breast cancer patients. East Afr Med J 87:147-150, 2010 23057289 10.4314/eamj.v87i4.62410

[21] Sung H, Ferlay J, Siegel RL, et al: Global cancer statistics 2020: GLOBOCAN estimates of incidence and mortality worldwide for 36 cancers in 185 countries. CA Cancer J Clin 71:209-249, 2021 33538338 10.3322/caac.21660

[22] Nzeyimana I, Nyirimodoka A, Ngendahayo E, et al: Diagnosis of advanced prostate cancer at the community level in Rwanda. Int Urol Nephrol 53:1977-1985, 2021 34191229 10.1007/s11255-021-02921-8

[23] Mchembe MD, Rambau PF, Chalya PL, et al: Endoscopic and clinicopathological patterns of esophageal cancer in Tanzania: Experiences from two tertiary health institutions. World J Surg Oncol 11:257, 2013 24094270 10.1186/1477-7819-11-257 PMC3850722

[24] Glasmeyer L, Mcharo RD, Torres L, et al: Long-term follow-up on HIV infected and non-infected women with cervical cancer from Tanzania: Staging, access to cancer-directed therapies and associated survival in a real-life remote setting. BMC Cancer 22:892, 2022 35971100 10.1186/s12885-022-09966-7 PMC9377112

